# Survival benefits from neoadjuvant treatment in gastric cancer: a systematic review and meta-analysis

**DOI:** 10.1186/s13643-022-02001-7

**Published:** 2022-07-04

**Authors:** Jianwen Hu, Yanpeng Yang, Yongchen Ma, Yingze Ning, Guowei Chen, Yucun Liu

**Affiliations:** 1 Department of General Surgery, Peking University First Hospital, Beijing, 100034 People’s Republic of China; 2 Endoscopy Center, Peking University First Hospital, Beijing, 100034 People’s Republic of China

**Keywords:** Gastric cancer, Preoperative treatments, Surgery alone, Systematic review, Meta-analysis

## Abstract

**Background:**

Surgery is the main treatment option for patients with local gastric cancer. However, surgery alone is usually not sufficient for stomach cancer patients, and combined therapies are recommended for these patients. In recent studies, some preoperative treatments have shown benefits. However, the treatment selection is still uncertain because previous studies failed to obtain a statistically significant difference between preoperative chemotherapy and preoperative chemoradiotherapy. Therefore, we plan to perform a systematic review and meta-analysis to compare the benefits among these preoperative treatments.

**Methods/design:**

This review includes randomized controlled trials with or without blinding as well as published studies, high-quality unpublished studies, full articles and meeting abstracts with an English context if sufficient results were provided for analysis. Data sources include the Cochrane Central Register of Controlled Trials, Embase, MEDLINE, major relevant international conferences and manual screening of references. Patients with a diagnosis of resectable primary gastric or EGJ adenocarcinoma (stage II or higher) who underwent surgery alone or preoperative treatment followed by surgery and who were pathologically confirmed as proposed by the AJCC 2017 guidelines without age, sex, race, subtypes of adenocarcinoma and molecular pathology limitations will be included. The following three interventions will be included: surgery alone, neoadjuvant chemistry followed by surgery and neoadjuvant chemoradiotherapy followed by surgery. All-cause mortality, overall survival (OS, the time interval from diagnosis to death) and/or progression-free survival (PFS, the time interval from diagnosis to disease progression or death from any cause) will be defined as major results of concern. The clinical and pathological response rate (according to RECIST and tumour regression score), R0 resection rate, quality of life and grade 3 or above adverse events (according to the National Cancer Institute Common Terminology Criteria for Adverse Events, NCI-CTCAE) will be defined as the secondary outcomes.

**Discussion:**

The aim of this systematic review is to compare the benefits of different preoperative treatments for patients with locoregional stomach cancer. This systematic review will improve the understanding of the relative efficacy of these treatment options by providing the latest evidence on the efficacy of various treatment options in the management of gastric cancer patients and may guide clinical practice.

**Systematic review registration:**

PROSPERO CRD4202123718

**Supplementary Information:**

The online version contains supplementary material available at 10.1186/s13643-022-02001-7.

## Background

### Epidemiology

As one of the most common tumours in the digestive tract, gastric cancer ranks fifth in incidence and fourth in cancer-related deaths. More than 720,000 gastric cancer patients were diagnosed in 2020 [1]. From the aspects of geography, high prevalence areas exist in Eastern Asia, and China has over half of the global gastric patients [1]. Moreover, the incidence rate of younger people (< 50 years) has markedly increased over the past recent years [2].

*Helicobacter pylori* is a carcinogen for stomach cancer, and approximately 90% of noncardia gastric cancer is attributed to this bacterium [3, 4]. In addition, dietary components also have a significant impact on the pathogenesis of stomach cancer, such as the intake of high salt, salt-preserved foods, smoking and the consumption of alcohol [5]. In addition, the risk of cardia gastric cancer can also be increased by obesity and gastroesophageal reflux disease (GERD) [6].

### Classification and staging system

More than 90% of stomach cancers are adenocarcinomas. Traditionally, stomach cancer can be divided into cardia and noncardia gastric cancer based on the location of the tumor [7]. A dramatic shift in the location of stomach cancer has occurred over the past decades. The incidence rate of noncardia stomach cancer has declined, mainly due to improved diet, food conservation and decreased *H. pylori* infection rates. However, the prevalence rate of cardia stomach cancer is rising, which may be a result of the high morbidity of GERD and obesity [8, 9].

In consideration of this transition, the eighth edition of the American Joint Committee on Cancer (AJCC) stomach cancer staging system has introduced a novel conception, namely, oesophagogastric junction (EGJ) cancer. Using this modification, tumours involving the EGJ with a centre located > 2 cm into the proximal stomach and cancers located in the cardia that do not involve the EGJ are staged as gastric carcinomas. Tumours involving the EGJ with an epicentre ≤ 2 cm into the proximal stomach are treated as oesophageal cancer [10]. Based on the purely anatomic location, EGJ tumours should be treated according to the corresponding guidelines.

The most commonly used staging system for gastric cancer is the eighth edition of the AJCC stomach cancer staging system. Stomach cancer is staged according to the T stage, N stage and M stage where T stage refers to the penetration depth of the tumour, N stage is the number of involved lymph nodes and M stage is the presence of metastases. However, up to 50% of patients present at an advanced stage, and nearly 80% of patients have lymph node metastases at diagnosis [11, 12]. Although the overall survival rate has improved in the past few years, the prognosis of gastric cancer is still poor [13]. The global 5-year survival rate of gastric cancer is approximately 20%, with the exception of 65% in Japan where screening is performed widely [14, 15].

### Description of the condition

Surgery is the main treatment option for patients with local gastric cancer. However, surgery alone is usually not sufficient for stomach cancer patients with locoregional disease. Combined therapy is required in this situation [16]. Interventions for preoperative treatments mainly include preoperative chemotherapy, preoperative chemoradiotherapy, preoperative sequential chemotherapy and chemoradiotherapy.

### Preoperative chemotherapy

Since the publication of the landmark Medical Research Council Adjuvant Gastric Infusional Chemotherapy (MAGIC) trial, neoadjuvant chemotherapy has been recommended for locoregional resectable stomach cancers [17]. This clinical trial advocates the use of epirubicin, cisplatin and 5-Fu (ECF) followed by surgery for the treatment of stage II or higher gastric cancer, resulting in a significant improvement in the benefit of progression-free survival [18]. Other studies have also indicated that preoperative chemotherapy can improve overall survival [19, 20]. Traditional perspectives of benefits from preoperative chemotherapy include downstaging of the tumour, which may increase the rate of R0 resection [21]. Moreover, preoperative chemotherapy may eradicate potential micrometastases [22]. Moreover, preoperative chemotherapy may eradicate potential micrometastases [23]. Good patient compliance is also superior to postoperative chemotherapy. The consequence of the recent phase II/III FLOT4 trial is also in accordance with the MAGIC trial. Investigators compared preoperative chemotherapy with fluorouracil, leucovorin, oxaliplatin and docetaxel (FLOT) in patients with resectable gastric cancer compared to standard ECF regimens. The phase III results of this trial show that FLOT is associated with significantly higher OS than ECF [24].

Surprisingly, the percentage of patients in the FLOT group with severe chemotherapy-related adverse events is the same as that in the ECF group, which is significantly reduced in the phase II trial [25]. Due to the considerable toxicity in the FLOT group, this regimen is recommended in select patients with good status. Although some studies have shown that the use of neoadjuvant systemic therapy has increased after the publication of these clinical trials, the most common treatment for locally advanced gastric cancer is still surgery alone [26].

### Preoperative chemoradiotherapy and preoperative sequential chemotherapy followed by chemoradiotherapy

Unlike studies on perioperative chemotherapy, there are few clinical trials on preoperative chemoradiotherapy. Several small, single-arm clinical trials have demonstrated a pathologic response after preoperative chemoradiotherapy for resectable gastric cancer [27, 28]. However, due to the lack of phase III randomized controlled trials showing the survival benefit of gastric cancer, the value of preoperative radiotherapy and chemotherapy for regional advanced gastric cancer is still unknown. Therefore, recommendations from panels for preoperative chemoradiation are mainly derived from trials of oesophageal and EGJ tumors [29]. Several studies have shown that preoperative sequential chemotherapy followed by chemoradiation and surgery generates a pathologic response in patients with localized advanced gastric cancer [30]. Perioperative chemotherapy prior to preoperative chemoradiation therapy is feasible and may be appropriate for select patients. However, this approach also needs to be further evaluated in phase III randomized clinical trials.

### Importance of the review

Up to 50% of stomach cancer patients have reached the advanced stage when diagnosed. Surgery alone for this situation is not enough. Combined therapies are recommended for them. Under modern studies, some preoperative treatments have shown benefits. But which one should we choose is still uncertain because previous studies failed to obtain a statistically significant difference between preoperative chemotherapy and preoperative chemoradiotherapy [31]. Therefore, we recommend a systematic review and meta-analysis to compare the benefits among these preoperative treatments.

### Objective

The objective is to compare the benefits of different preoperative treatments for patients with locoregional stomach cancer.

## Methods/design

### Criteria considered in this review

#### Study types

This review will only include randomized controlled trials with or without blinding. As some studies include participants with symptomatic disease, the blinding of participants or use of placebo control can be difficult to implement; hence, they are not mandatory requirements. We will also include both published and high-quality unpublished studies as well as full articles and meeting abstracts with an English context if sufficient results for analysis are provided.

#### Types of participants

Patients with a diagnosis of resectable primary gastric or EGJ adenocarcinoma (stage II or higher) who underwent surgery alone or preoperative treatment followed by surgery and who were pathologically confirmed as proposed by the AJCC guidelines without age, sex, race, adenocarcinoma subtype and molecular pathology limitations will be included.

#### Intervention types

The following three interventions will be included: surgery alone, neoadjuvant chemotherapy followed by surgery and neoadjuvant chemoradiotherapy followed by surgery. Subgroup analyses will be conducted according to the neoadjuvant treatments in the included trials. Planned surgery alone will be defined as the control arm, and neoadjuvant treatments followed by planned gastrectomy will be defined as the intervention arm. We will also compare the benefits of different preoperative treatments for patients with locoregional stomach cancer.

Chemotherapy is defined as the administration of cytotoxic drugs and includes but is not limited to 5-FU, cisplatin, oxaliplatin, carboplatin, docetaxel, paclitaxel and irinotecan given as single agents or in combination.

Radiotherapy refers to applying radioactive substances to generate internal injuries to treat diseases, such as cancers. With a history of approximately 100 years, radiation therapy has been developed into a mature system.

Due to the development of computers, three-dimensional conformal radiotherapy (3-DCRT), intensity-modulated radiotherapy therapy (IMRT) and image-guided radiotherapy therapy (IGRT) have achieved the delivery of high-dose radiation to tumours, increased the efficiency of radiotherapy and increased the preservation of normal tissues.

### Outcome measure types

#### Primary outcomes

All-cause mortality, overall survival (OS, the time interval from diagnosis to death) and/or progression-free survival (PFS, the time interval from diagnosis to disease progression or death from any cause) will be defined as the major results of concern.

#### Secondary outcomes

The clinical and pathological response rate (according to RECIST and tumour regression score), R0 resection rate, quality of life and grade 3 or above adverse events (according to the National Cancer Institute Common Terminology Criteria for Adverse Events, NCI-CTCAE) will be defined as the secondary outcomes.

### Research identification retrieval methods

#### Database searches

All published and unpublished RCTs in English will be identified by conducting a literature search.

The following electronic databases will be searched:

1. Cochrane Central Register of Controlled Trials (CENTRAL, latest issue; Appendix 1)
2. Embase (1988 to date of search; Appendix 2)
3. MEDLINE (1966 to data of search; Appendix 3)

We will also search PubMed before completing the review to obtain records that fail to be accessed through MEDLINE.

#### Searches using other resources

The reference lists of all primary studies and review articles for other references that may have been missed during our original electronic searches will be checked. The necessary authors will be contacted to identify the research, and they will be asked to identify other relevant published and unpublished research. We will search clinical trial registers for unpublished data and consider sending a comprehensive list of relevant articles to the first authors included in the research report and asking them if they know of any other studies that may be relevant. Corrigenda or retractions of eligible studies will be searched on PubMed, and we will report relevant data in the review. Published abstracts or reports from the following major relevant international conference proceeding from 2010 onwards will be searched:

1. American Society of Clinical Oncology (ASCO; https://www.asco.org)
2. European Society for Medical Oncology (ESMO; https://www.esmo.org)
3. International Gastric Cancer Congress (IGCC; http://www.igca.info/igcc/index.html)

#### Grey literature databases

We will identify prospective and ongoing studies by searching the following prospective trial registers:

1. International Standard Randomized Controlled Trial Number Registry (www.controlled-trials.com)
2. US National Institutes of Health (www.clinicaltrials.gov)
3. US National Cancer Institute (www.cancer.gov/clinicaltrials/search)
4. International Clinical Trials Registry Platform (www.who.int/trialsearch)

#### Manual search

We will check the reference lists of the included research, previous systematic reviews and key textbooks through a manual search to determine further research reports.

### Data collection and analysis

#### Screening of studies

The titles and abstracts of all potential studies will be screened by two review authors (Jianwen Hu and Yanpeng Yang) and marked as “retrievable” (eligible, potentially eligible or unclear) or “not retrieved.” The full text of publications or research reports will be researched, and the full text will be screened separately (Jianwen Hu and Yanpeng Yang) in the “Features of Excluded Studies” table to determine the studies to be included. The reasons for excluding unqualified studies will be recorded in the “Features of Excluded Studies” table. To ensure that each study rather than each report becomes the focus of the review, multiple reports of the same study will be identified, eliminated and organized. The identification and screening criteria for multiple reports of the same study are as follows: (1) reports with more detailed and complete data are preferred, (2) reports with duplicate data are reserved for either, (3) reports with more complete follow-up data are preferred, (4) reports corrected by the journal will cite the results of the updated version and (5) priority will be given to citing data corresponding to reports of higher quality published. Any nonconformities will be resolved through discussion with a third member of the team (Yongchen Ma) or consultation with the fourth reviewer (Yingze Ning) if necessary. The selection process will be documented in detail to generate a flowchart and to provide a list of the characteristics of excluded studies. Two authors (Guowei Chen and Yucun Liu) will be responsible for determining the analysis strategies and the guidance and supervision during the analysis process.

#### Data extraction and management

A standard data collection form will be used to collect characteristic and outcome data that have been tested in at least one study included in this review. Two independent review authors (Jianwen H and Yanpeng Yang) will obtain the following research characteristics from the included studies and indicate them in the “Included Studies” table:

1. Methods: study design, study duration, run-in period, number of study centres, number of study locations, study setting, withdrawals and data
2. Participants: number, mean age, age range, sex, stage of disease, presenting symptoms, included criteria, excluded criteria and diagnostic criteria
3. Interventions: radiation dose/fractionation, type of radiotherapy techniques, use of concurrent chemotherapy with radiotherapy, types of chemical drugs and dosage of chemical drugs
4. Comparison: all-cause mortality, overall survival, progression-free survival, clinical response rate, pathological response rate, R0 resection rate, quality of life and grade 3 or above adverse events of different preoperative treatments for patients
5. Outcomes: specify and collect primary and secondary results as well as report time points
6. Items requiring attention: study funding and authors with obvious conflicts of interest

If the reporting method of the result data is not available, we will indicate it in the “Features of the included study” table. We will resolve the differences through consensus or by the third author (Yongchen Ma). The data from the collection form will be copied into Review Manager 5.3 (Review Manager 2014) by a review author (Yingze Ning). A comment author (Yongchen Ma) will double-check the data to make sure it is centred correctly. The second commentary author (Yanpeng Yang) will randomly check the accuracy of the research features based on the research report.

For time to event (survival) data, the logarithm of the hazard ratio and standard error from the study reports will be extracted; if the hazard ratio is not reported, the logarithm of the hazard ratio will be estimated using previously published methods [28, 29].

For dichotomous outcomes (response rates and adverse events), the number of participants who experienced the relevant outcome in each treatment group and the number of participants evaluated at the end point will be extracted to estimate the risk ratio with a 95% confidence interval.

For continuous outcomes (duration of response), the final value and standard deviation of the relevant outcome as well as the number of participants evaluated in each treatment group at the endpoint will be extracted to estimate the average difference between the treatment groups and their standard error. If a different scale is used to report the results, we will estimate the difference in standardized means.

#### Risk assessment of bias in included studies

Two authors (Jianwen Hu and Yanpeng Yang) will independently assess the risk of bias for each study using the criteria outlined in the Cochrane Handbook for Systematic Reviews of Interventions [32]. We will resolve any differences by consultation or by involving a third author (Yongchen Ma). We will assess the risk of bias based on the following areas:

1. Selection bias: random sequence generation;
2. Performance bias: allocation concealment and blinding of participants and personnel
3. Detection bias: blinding of outcome assessment
4. Attrition bias: incomplete outcome data
5. Reporting bias: selective outcome reporting
6. Other bias:
7. Was the sample size predefined and was the target accrual number reached?
8. Was there unplanned interim analysis?
9. Was radiological tumour response assessed by trial investigators or by independent/blinded radiologists?
10. Were baseline characteristics balanced??

#### Bias assessment for review

The review will be based on this plan, and any differences from the plan will be reported in the “Differences between plan and review” section of the plan.

#### Treatment effect measures

We will use 95% confidence intervals (CIs) to analyse binary data as risk ratio (RRs), and we will analyse continuous data as the mean difference (MD) or standard mean difference (SMD), ensuring that the higher scores of consecutive results have the same meaning for a particular result.

Health-related quality of life scores will be used as continuous data (rather than arbitrarily categorized), and we will follow the recommendations in the Cochrane System Intervention Review Manual to express these data as SMD with 95% CI.

A positive SMD will be considered as having a better effect on the quality of life, while a negative SMD negative will be considered as having a beneficial effect on mental health and cancer-related symptoms. We will only conduct a meta-analysis if the treatment, participants and underlying clinical problem are sufficiently similar to obtain a significant combined analysis. Considering the substantial heterogeneity of the surgical results, we will use the inverse variance random-effects model. If the continuous results are biassed, we will apply a natural logarithmic transformation to improve the normality of the data before the combined analysis.

Clinical trial researchers often report that the median and interquartile ranges lead to skewed data [33]. If and when we encounter this situation, we will discuss the skewed data and consider the implications of this finding. If we suspect that the data are skewed, we will carefully interpret the results and consider sensitivity analysis, except for those studies with skewed data. Otherwise, we will provide a narrative summary.

#### Unit of analysis issues

We will combine the groups to create a single pairwise comparison for studies with multiple interventions if it is clinically meaningful. For example, for a clinical trial with three arms (arm A, neoadjuvant chemotherapy A plus surgery; arm B, neoadjuvant chemoradiotherapy B plus surgery; and arm C, surgery alone), we will combine the results of arms A + B and compare the combined results of arms A + B against the results of arm C. We will analyse each eligible study for the potential unit of analysis errors, such as reporting multiple observations for the same outcomes. If it is impossible to extract data for a single observation for each patient, it will be excluded from the meta-analysis.

#### Addressing missing data

If there is a lack of data and the study is only published in abstract form or missing numerical results, we will contact the relevant trial authors to verify the key research features and results. If we do not receive the required information, we will use the method described in the Cochrane System Intervention Review Manual to calculate or estimate SD based on other values and information in the paper.

When necessary, we will use the methods of Parmar et al. [34] and Williamson et al. [35] to estimate the unreported hazard ratio and its variances from the long-term chi^2^ or *P* value, the median occurrence of the time-to-event outcomes and survival rate at a given point. Similarly, the median time-to-event outcomes and survival rate of unreported events can be extrapolated from the Kaplan–Meier survival curve. If there is insufficient information to impute the treatment effect estimates using the methods proposed above, we may reconstruct survival time data from the published Kaplan–Meier (KM) survival curves using the methodology of Guyot et al. [36] if the number at risk and the number of events at each time point are available. Thus, we can estimate the HR and its 95% confidence interval from the reconstructed KM data. If estimates are not possible, we will not include such data in the meta-analysis.

#### Heterogeneity assessment

Cochran’s *Q* test and the Higgins *I*^2^ statistic will be used to measure the heterogeneity among the included studies. In addition, we will also assess the heterogeneity by visually inspecting the forest plots and conducting tests to estimate the overall variation across studies (by using the chi^2^ test) to assess whether there is a good overlap in CI.

The heterogeneity is considered significant if the *I*^2^ is greater than 50% or if there is a low *P* value (< 0.10) in the chi^2^ test for heterogeneity.

#### Assessment of reporting biases

If more than 10 studies can be aggregated, a funnel chart will be created and examined to explore possible publication bias, and Egger’s test will be used to ascertain the statistical significance of reporting bias [37]. *P* < 0.05 will be considered as a statistically significant reporting deviation.

#### Data integration

We will use a random-effects model for the meta-analysis and further investigate the sources of heterogeneity when necessary based on the recommendations from the Cochrane Handbook for Systematic Reviews of Interventions using Review Manager 5.3. Manager 5.3 will be used to record and analyse the data. To test the robustness of our findings, a fixed-effects model will be used to conduct a sensitivity analysis of the main results regardless of the method selected. If there is a difference between the two models, we will provide two results at the same time. Otherwise, we will only present the results of the random-effects model. If we are unable to pool data statistically using meta-analysis, we will conduct a narrative synthesis of results adhering to the Synthesis Without Meta-analysis (SWiM) guideline [38].

For time-to-event data, we will use general inverse variance to summarize the hazard ratio. If a substantial number of studies exhibit nonproportionality of hazards in the treatment comparisons, we will implement the Cox time-dependent covariate model to estimate the treatment effects at 3 or 5 years. To do this, we will construct the individual patient data from information extracted from the Kaplan–Meier curve using the method of Guyot et al. [36], which has been shown to have a high degree of reproducibility with reasonable accuracy for estimating the HR if at least the numbers at risk or the total number of events are reported. The Schoenfeld test [39] for nonproportionality may then be implemented on the reconstructed KM data. When the assumption of proportionality is violated for the treatment variable, a Cox model with treatments as the time-dependent covariate may be generated to estimate the HR at 3 and 5 years [40]. The hazard ratio will be calculated from the number of events. If possible, the hazard ratio will be derived from the 3-year actuarial survival rate; otherwise, the 2-year actuarial survival rate will be used.

For dichotomous outcomes, we will calculate the odds ratio (OR) or risk ratio (RR) applicable to each study and obtain aggregate estimates from these studies. We will analyse the data based on the number of events and the number of participants evaluated in the intervention and comparison groups, and we will use these data to calculate the RR and 95% confidence interval (CI).

If all studies measure the results on the same scale, the mean difference (MD) between the treatment groups will be summarized at the end of follow-up for continuous results. If more than one study use different tools to measure the same results, the inverse variance method will be used to calculate the standardized mean (SMD) and 95% CI. The continuous results will be summarized based on the mean, standard deviation (SD) and the number of participants in the intervention and comparison groups to calculate the mean difference between the treatment group and the associated 95% CI. If there are no separate group data for the reported MD, we will use it to report the results of the study.

We will combine the treatment groups as appropriate to avoid multiplicity problems if any study has multiple treatment groups. We will perform a narrative synthesis of the results if we cannot count the aggregated data for meta-analysis. We will describe the main results according to the intervention category according to the main type or goal of the identified intervention or according to both. Based on the assembled data, we may also explore the possibility of providing data by population. In the data category, we will explore the main comparisons of reviews.

#### “Summary of findings” table

A “Summary of findings” table will be created with the following outcomes:

1. Overall survival
2. Progression-free survival period to progression
3. Recurrence-free survival time
4. Objective response rates
5. Duration of response for each symptom
6. Adverse events
7. Quality of life
8. Serious adverse events
9. Rate of R0 resection

Five considerations (research limitations, consistency of effect, imprecision, indirectness and publication bias) of GRADE will be used to assess the quality of evidence-based studies that provide data for the meta-analysis of each outcome. The quality is classified as high, medium, low or very low. The methods and recommendations described in Section 8.5 and Chapter 12 of the Cochrane System Intervention Guide and GRADEpro GDT software (GRADEpro GDT) will be used.

We will consider whether there are other comments that are not included in the meta-analysis and indicate whether they support or oppose the information in the meta-analysis.

#### Subgroup analysis and heterogeneity investigation

The following factors will be investigated through subgroup analysis:

1. Effects of neoadjuvant therapy.
2. Effects of age, sex, race, region, pathological differences and different adjuvant treatment programmes due to possible obvious heterogeneity or inconsistency will be used to identify possible sources.
3. Studies that include chemotherapy versus studies that did not include chemotherapy.
4. Radiotherapy administered at a biologically effective dose (using a tumour alpha/beta ratio of 10) greater than 39 Gy or less.
5. Studies that compare the high-to-moderate risk of bias versus the low risk of bias.
6. Choice of a chemotherapy regimen in the control or experimental arms or both arms (in case of studies using combinations of chemotherapy).
7. Studies with a heterogeneous population, including Asian and African American participants, versus studies with a homogeneous population.
8. Study population according to geographical region.
9. Men versus women.
10. Furthermore, we will establish a meta-regression model, screen out the influencing factors of heterogeneity, perform a subgroup analysis based on the factors and compare the changes in heterogeneity before and after the subgroup analysis.

The following results will be included in subgroup analyses.

1. Response rates of bleeding
2. Response rates of pain
3. Response rates of obstruction

The formal Altman interaction test will be used to test subgroup interactions [41].

#### Sensitivity analysis

The following sensitivity analyses will be performed:

1. Fixed-effect model versus the random-effects model
2. Studies without data imputation versus studies with data imputation

## Conclusions

Our conclusions will be based solely on the quantitative or narrative comprehensive research results in this review. We will make the readers aware of the focus of future research and the uncertainties in the field.

## Discussion

Recent studies have reported that some preoperative treatments have shown benefits for gastric cancer. However, treatment selection remains uncertain because previous studies failed to obtain a statistically significant difference between preoperative chemotherapy and preoperative chemoradiotherapy. Therefore, this systematic review and meta-analysis will compare the benefits of these preoperative treatments and provide theoretical guidance for patients with gastric cancer to select an optimal treatment.

The systematic review and meta-analysis proposed in this agreement will be reported in accordance with the reporting guidelines provided in the Preferred Reporting Items for Systematic Reviews and Meta-Analyses extension for Protocols (PRISMA) statement [42]. The checklist of PRISMA declarations will serve as the quality control criteria for references, and each included article will be scored for evaluation (Additional file 1: Table S1). Any changes or modifications made in the agreement will be summarized and reported in the final document.

Our research has advantages and limitations. This protocol considers research heterogeneity, sensitivity, publication bias and other issues, and the design is reasonable. There are many major resolution events and secondary ending events that need to be considered, and we will merge them according to the final literature search results to present the research content concisely. However, there may be problems, such as fewer research results and inconsistent controls, which need to be further determined based on the search results. If there is a problem of high heterogeneity, we will perform a subgroup analysis to analyse the source of the heterogeneity and clarify.

This systematic review will improve the understanding of the relative efficacy of these treatment options by providing the latest evidence on the efficacy of various treatment options in the management of gastric cancer patients and may guide clinical practice. Therefore, this systematic review will benefit a wide audience, including gastric cancer patients, oncologists, policymakers, insurance companies, and researchers working in the field of oncology.

### Supplementary Information

**Additional file 1 : Table S1.** PRISMA 2020 Checklist.

## Data Availability

Not applicable.

## Appendix

### Appendix 1

CENTRAL search strategy

### Appendix 2

MEDLINE search strategy

### Appendix 3

Embase search strategy
